# Genome‐wide diversity analysis suggests divergence among Upper Guinea and the Dahomey Gap populations of the Sisrè berry (Syn: miracle fruit) plant (*Synsepalum dulcificum* [Schumach. & Thonn.] Daniell) in West Africa

**DOI:** 10.1002/tpg2.20299

**Published:** 2023-01-20

**Authors:** Dèdéou A. Tchokponhoué, Enoch G. Achigan‐Dako, N'Danikou Sognigbé, Daniel Nyadanu, Iago Hale, Alfred O. Odindo, Julia Sibiya

**Affiliations:** ^1^ School of Agricultural, Earth and Environmental Sciences University of KwaZulu‐Natal Pietermaritzburg South Africa; ^2^ Laboratory of Genetics, Biotechnology and Seed Science (GBioS), School of Plant Sciences University of Abomey‐Calavi Abomey‐Calavi Republic of Benin; ^3^ Ecole d'Horticulture et d'Aménagement des Espaces Verts Université Nationale d'Agriculture Kétou Republic of Benin; ^4^ World Vegetable Center, East and Southern Africa Arusha Tanzania; ^5^ Cocoa Research Institute of Ghana (CRIG) Akim Tafo Ghana; ^6^ Department of Agriculture, Nutrition, and Food Systems University of New Hampshire Durham NH USA

## Abstract

Although *Synsepalum dulcificum* is viewed as one of the most economically promising orphan tree crops worldwide, its genetic improvement and sustainable conservation are hindered by a lack of understanding of its evolutionary history and current population structure. Here, we report for the first time the application of genome‐wide single nucleotide polymorphism genotyping to a diverse panel of *S. dulcificum* accessions to depict the genetic diversity and population structure of the species in the Dahomey Gap (DG) and Upper Guinea (UG) regions to infer its evolutionary history. Our findings suggest low overall genetic diversity but strong population divergence within the species. Neighbor‐joining analysis detected two genetic groups in the UG and DG regions, while STRUCTURE distinguished three genetic groups, corresponding to the UG, Western DG, and Central DG regions. Application of Monmonier's algorithm revealed the existence of a barrier disrupting connectivity between the UG and DG groups. The Western DG group consistently exhibited the highest levels of nucleotide and haplotype diversities, while that of the Central DG exhibited the lowest. Analyses of Tajima's *D*, Fu's *F*s, and Achaz Y* statistics suggest that while both UG and Central DG groups likely experienced recent expansions, the Western DG group is at equilibrium. These findings suggest a geographical structuring of genetic variation which supports the conclusion of differential evolutionary histories among West African groups of *S. dulcificum*. These results provide foundational insights to guide informed breeding population development and design sustainable conservation strategies for this species.

## Core Ideas

- Genome wide, SNP‐based diversity is investigated for the first time in *Synsepalum dulcificum*.
- There is low genetic diversity but clear differentiation among the distinct West African populations of the species.
- At least two major gene pools are detected within West Africa, in alignment with ecological demarcations.
- The major gene pools exhibit differentiated evolutionary histories.
- Foundational knowledge is generated to guide rational germplasm collection and breeding activities for *S. dulcificum*.

**Abbreviations**
AMOVA: analysis of molecular variance
CDG: Central Dahomey Gap
DG: Dahomey Gap
DNA: deoxyribose nucleic acid
MAF: minor allele frequency
RAPD: random amplified polymorphism DNA
SNP: single nucleotide polymorphism
SSR: single sequence repeat
UG: Upper Guinea
WDG: Western Dahomey Gap

## 1 INTRODUCTION

*Synsepalum dulcificum* (Schumach. & Thonn.) Daniell [Syn. *Richardella dulcifica* (Schumach. & Thonn.) Baehni] is a slow‐growing evergreen fruit tree species (Tchokponhoué et al., 2018) in the Sapotaceae family. A long living monoecious species that rarely reaches a height exceeding 8 m (Tchokponhoué et al., 2020), *S. dulcificum* produces red berries known as Sisrè that contain recalcitrant seeds (Tchokponhoué et al., 2019). The species is sometimes referred to as the miracle plant, but that common name is shared by unrelated species, including *Bryophyllum pinnatum* (Lamarck) (Kamil et al., 2021), *Gymnema sylvestre* (Retz) Schult (Khanna et al., 2011), and others. To avoid confusion, *S. dulcificum* will hereafter be referred to as the Sisrè berry plant (Tchokponhoué et al., 2021).

To date, the Sisrè berry plant is the only known natural source of miraculin (Kurihara & Beidler, 1968), a taste‐modifying glycoprotein contained in the mucilaginous flesh of the fruit. Coupled with the novel sweetening property of miraculin, the Sisrè berry's high micronutrient and antioxidant contents (Du et al., 2014) have led to its use in a wide range of culinary and pharmaceutical applications, the latter including both diabetes control and the enhancement of taste perception in patients with cancer (Buckmire & Francis, 1978; Wilken & Satiroff, 2012). The seed oil has been shown to improve hand motor skill and prevent hair breakage in women (Del Campo et al., 2017; Gorin et al., 2018), while various other parts of the plant (e.g., roots, leaves, and bark) are excellent sources of numerous health‐promoting phytochemicals, such as vanillic acid, epi‐syringaresinol, β‐sitosterol, and lupeol (Chen et al., 2010; Masson, 2014). On the international market, dried Sisrè berry fetches an astounding price of USD $2500 per kg of pure powder (Link 1, Link 2). Given the diversity of its functional uses and its high economic value, *S. dulcificum* stands as one of the most significant and promising berry crops worldwide, even moreso in light of its recent formal authorization in the European Single Market (Turck et al., 2021).

Compared with moreheavily invested berry crops, however, such as strawberry (*Fragaria* × *ananassa*) (Barbey et al., 2020; Denoyes et al., 2016; Nelson et al., 2021), blueberry (*Vaccinium corymbosum*) (Die & Rowland, 2013; Qi et al., 2021), cranberry (*Vaccinium macrocarpon*) (Covarrubias‐Pazaran et al., 2016; Diaz‐Garcia et al., 2021), and blackberry (*Rubus* spp.) (Foster et al., 2019; Garcia‐Seco et al., 2015), all of which benefit from long breeding histories and extensive genomic resources, Sisrè berry is an orphan crop with no systematic history of breeding or readily available genetic resources or information to facilitate its improvement. Despite the very recent publication of a reference genome for *S. dulcificum* (Yang et al., 2022), basic information on genetic diversity within the species, though fundamental to implementing an effective breeding program, is scanty and limited to a single low‐resolution assessment of a geographically narrow collection (Chibuzor et al., 2017). Although three distinct phenotypic groups are reported in the species in West Africa (Tchokponhoué et al., 2020), such groups are yet to be corroborated by molecular evidence. To date, the only reported molecular evaluation of diversity in the species (Chibuzor et al., 2017) is based on RAPD markers, a platform that today is considered outmoded in light of the advent and now common application of reduced‐representation library sequencing approaches, such as genotyping‐by‐sequencing (Elshire et al., 2011), restriction site‐associated DNA sequencing (Miller et al., 2007), Diversity Array Technology Sequencing (Sansaloni et al., 2011), and others. The relative advantage of such methods lies in their ability to efficiently generate genome‐wide sets of high‐quality single nucleotide polymorphisms (SNPs) suitable for high resolution molecular diversity assessments, even in crops currently lacking a reference genome (Feng et al., 2019; Melo et al., 2016; Poland & Rife, 2012). Being less prone to mutation compared to other types of markers (e.g., SSR markers), SNPs are also more stable, thereby offering better insight into evolutionary histories (Carrasco et al., 2018; Wei et al., 2021). Apart from the limitations of the RAPD platform, however, the study's conclusions were based on only 40 individuals sampled exclusively in Southern Nigeria, whereas the species has a much larger natural distribution, spanning West and Central Africa. In sum, there is a clear opportunity and need to bring modern sequencing‐based assessment methods to bear on a much‐expanded sampling of the species, not only in terms of geographical coverage but also numbers, to achieve the resolution necessary for reliable diversity and population structure assessment.

The Sisrè berry plant naturally occurs in the West African rainforest, a dense tropical humid vegetation block that extends, west to east, from Guinea to the Democratic Republic of the Congo (Leal, 2004). The forest vegetation in this region experienced climatic fluctuations during the Holocene that ultimately induced its disruption into three ecologically distinct blocks: two forested blocks, (western Upper Guinea and eastern Lower Guinea) and one intervening savannah block known as the Dahomey Gap (DG) (White, 1983). Currently perceived as an open savanna, the DG successively experienced (i) a shift from its original semi‐evergreen forest state to a Sudano‐Guinean savannah zone during the dry Glacial Stages (8400–4500 cal. year BP); (ii) a forest re‐establishment, a consequence of rewetting under the environmental conditions of the Late Interglacial and early Holocene (3300–1300 cal. year BP); and (iii) an establishment of the persisting open savannah seen today (Dupont & Weinelt, 1996). It is widely acknowledged that past climatic fluctuations, such as those observed in the DG, can drive significant changes in plant species distribution, diversification, evolutionary dynamics, and speciation (Demenou et al., 2018; Duminil et al., 2015; Hagen et al., 2021; Plana et al., 2004; Welker, 2017). The extent of such influences has been rarely explored under tropical conditions, however (Hagen et al., 2021; Teixeira et al., 2021). The current distribution of *S. dulcificum* in these three ecologically distinct West African blocks offers a unique opportunity to gain insight into the effects of past climate changes on the genetic variation and population reconfiguration of a slow‐growing tropical tree species.

**Core Ideas**

- Genome wide, SNP‐based diversity is investigated for the first time in *Synsepalum dulcificum*.
- There is low genetic diversity but clear differentiation among the distinct West African populations of the species.
- At least two major gene pools are detected within West Africa, in alignment with ecological demarcations.
- The major gene pools exhibit differentiated evolutionary histories.
- Foundational knowledge is generated to guide rational germplasm collection and breeding activities for *S. dulcificum*.

By applying genome‐wide, reduced representation sequencing to a large and geographically diverse collection of *S. dulcificum* germplasm, this study seeks to understand the basic population structure of the species, particularly as it relates to the Upper Guinea–Dahomey Gap ecological divergence. Beyond this, the objective of the study is to infer the evolutionary history of the species and shed light on the current patterns of genetic diversity to guide conservation and breeding efforts.

## 2 MATERIALS AND METHODS

### 2.1 Study area

**FIGURE 1:**
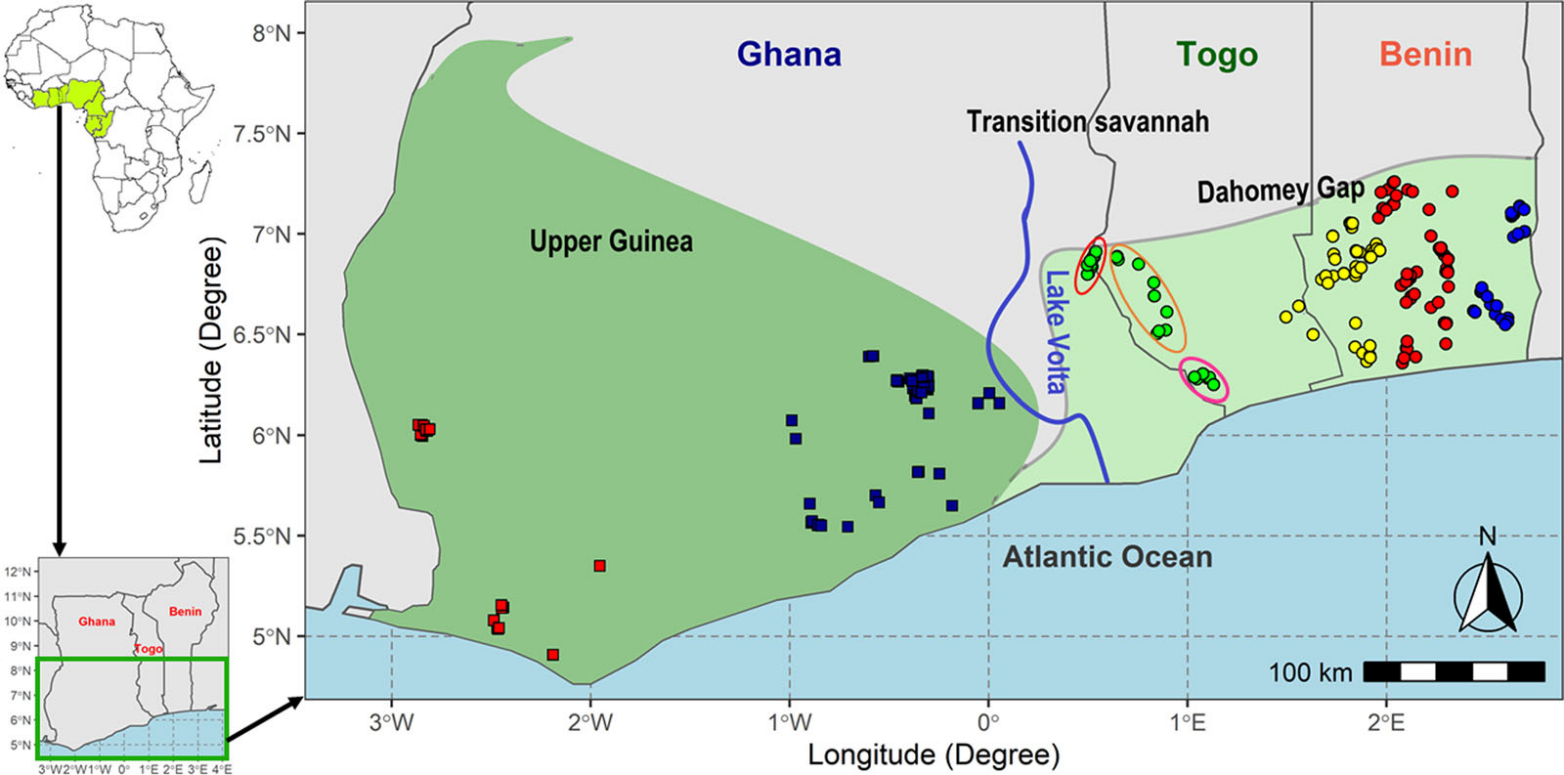
Map showing the locations of the Sisrè berry plant (*Synsepalum dulcificum*) accessions sampled in the Upper Guinea (UG) and Dahomey Gap (DG) blocks of the West African tropical rainforest for this study. The locations of individual accessions are indicated by squares (UG block) or circles (DG block). The two UG populations are the Western (red) and Eastern (blue) populations. The four DG populations are the Volta (green), Mono (yellow), Zou (red), and Oueme (blue) populations. The three ellipses, west to east, indicate the Volt1, Volt2, and Volt3 sub‐populations, respectively. In the inset map of Africa, countries shaded light green encompass the entire distribution range of the species

This study specifically focused on the Upper Guinea (UG) and DG blocks of the West African rainforest (Figure 1), as these two areas were previously implicated in constituting the potential center of origin and diversity of the Sisrè berry plant (Swenson et al., 2008; Tchokponhoué et al., 2020). Also known as the Guinean forest block, the UG block extends from Guinea to eastern Ghana (Leal, 2004) and is dominated by semi‐evergreen and evergreen rainforests experiencing annual rainfalls of 1600–2000 and 2000–4551 mm, respectively (Duminil et al., 2013). The DG is a ca. 200 km broad savannah corridor created during the late Holocene, following abrupt changes in climate 4500 and 3400 cal. years BP (Salzmann & Hoelzmann, 2005). The annual rainfall in the DG is between 1000–1200 mm. Indicated as a biogeographical barrier between the Upper and Lower Guinea blocks, the DG encompasses the coasts of Benin, Togo, and Ghana and is the most significant distributional disjunction for forest taxa in West Africa (Fuchs & Bowie, 2015).

### 2.2 Tree sampling

From September 2016 to August 2020, a total of 322 individual trees (hereafter, accessions) of *S. dulcificum* were sampled from six populations (Table 1) spread across the UG and DG blocks, all within the political boundaries of the countries of Benin, Togo, and Ghana. For the purposes of sampling, a population of Sisrè berry plant was defined according to Tchokponhoué et al. (2020) as a set of individuals possibly interbreeding and randomly distributed within a defined agroecosystem. To be considered distinct, two adjacent populations of *S. dulcificum* had to be separated not only by a minimum distance of 15 km but also by a natural barrier, such as the rivers separating adjacent populations Mono, Zou, and Oueme in Benin. Two of the six populations, namely the Volta and Mono populations, were cross‐country populations: The “Volta” population extends from western Togo to the Volta region of Ghana, and the “Mono” population stretches from western Benin to eastern Togo.

**TABLE 1:** Distribution of Sisrè berry plant (*Synsepalum dulcificum*) accessions sampled in this study, grouped by ecological region and population

Ecology	Country	Population	Population code	Latitudinal range	Longitudinal range	Altitude range (m)	Sample size
Upper Guinea				N 05°20′59.6″─N 06°23′34.7″	W 002°51′55.9″ ─W 000°03′12.7″	9─579	110
	Ghana	Western	WEST	N 05°20′59.6″ ─N 06°03′07″	W 002°51′55.9″ ─W 001°57′17.6″	9─276	30
		Eastern	EAST	N 05°39′00.3″ ─N 06°23′34.7″	W 000°59′23.4″ ─W 000°03′12.7″	37─579	80
Dahomey Gap				N 06°14′54.8 ─N 07°15′30.4″	E 000°29′45.6″ ─E 002°41′39.9″	−2 to 570	212
	Ghana and Togo	Volta	VOLT	N 06°14′54.8 ─N 06°50′45.8″	E 000°29′45.6″ ─E 001°07′50.7″	34─570	40
	Togo and Benin	Mono	MON	N 06°21′49.59″ ─N 07°03′06.8″	E 001°29′40.7″ ─E 001°57′58.5″	−2 to 220	74
	Benin	Zou	ZOU	N 06°21′28.4′' ─N 7°15′30.4″	E 001°57′38.1″ ─E 02°20′9.559″	11─247	67
		Oueme	OUE	N 06°32′46.6″ ─N 07°08′16.6″	E 002°26′17.2″ ─E 002°41′39.9″	11─138	31

The number of accessions sampled per population ranged from 30 to 74 (Table 1). Within a population, sampled individuals were at least 100 m from each other to reduce the risk of collecting closely related individuals. Each sampled accession was georeferenced (Longitude, Latitude, Altitude) using a Garmin eTrex10® GPS (Table S1).

### 2.3 Leaf tissue sampling and genomic data generation

For each accession, three to five young and visually pest‐free leaf samples were collected and directly dried using silica gel to preserve the DNA for further use. Organized into collection kits consisting of racks of 96 tubes, 15–20 mg of silica‐gel dried leaf tissue from each accession was shipped to SEQART AFRICA (Nairobi, Kenya), formerly Integrated Genotyping Support and Service (IGSS), a branch of Diversity Arrays Technology Pty. Ltd. (Canberra, Australia), for DNA extraction, library preparation, and genotyping. Following DNA extraction using the company's in‐house protocol (Box S1), complexity reduction was achieved using a standard two enzymes system (*Pst1* and *MseI*). Genotyping based on high‐density sequencing on the Illumina Hiseq 250 platform was accomplished via the DArTseq™ pipeline, with a target of 2,500,000 reads per sample (Sansaloni et al., 2011).

### 2.4 Bioinformatics and genetic analyses

Raw read processing and SNP calling were done using Diversity Array Technology's propriety analytical pipelines (Walters et al., 2020), incorporating technical replicates as a measure of reproducibility at each locus. The generated SNPs were further quality‐filtered prior to their use in downstream analyses. First, the “dartR” package (Gruber et al., 2018) (V 1.9.9) was employed to filter out low‐quality putative SNPs that (i) deviated from Hardy–Weinberg equilibrium at a threshold of 5%; (ii) had a call rate <85% across the population; (iii) had a minor allele frequency (MAF) <5%; and (iv) had an average repeatability of alleles at a locus (reproducibility rate) <95% (Canales et al., 2021; Kui et al., 2020; Park & Donoghue, 2019; Pereira et al., 2020; Tomar et al., 2019). Missing cells in the genotyping matrix obtained after this first round of filtering were imputed based on the Ensemble Method algorithm as implemented by the “Optimal Imputation V. 1.0.5” plugin on the KDcompute platform (https://kdcompute.seqart.net/kdcompute/plugins). The resultant imputed matrix was then re‐filtered based on MAF (loci with MAF <5% culled) to obtain the final genotyping matrix for use in all downstream analyses. Finally, the “dartR” package was used to generate file formats suitable for analyses in other relevant packages.

#### 2.4.1 Genetic diversity, population divergence, and isolation by distance

Genetic diversity in the species was characterized by computing usual diversity indices, including observed heterozygosity (*H*_o_), expected heterozygosity (*H*_s_), inbreeding coefficient (*F*_IS_), and total gene diversity (*H*_t_), all using the *gl.basic.stats()* function in the “dartR” package. Each statistic was tested for significant departure from 0 with the function *overallTes*t*()* in the “strataG” package (Archer et al., 2017). *H*_o_, *H*_s_, *F*_IS_, and allelic richness (*A*_r_) were also computed within populations to assess population‐to‐population differences in genetic diversity. *H*_o_ and *H*_s_ were computed with the functions *gl.report.heterozygosity()* in “dartR”, *F*_IS_ with the function *basic.stats()* in the “hierfstat” package (Goudet et al., 2015), and *A*_r_ with the function *allel.rich()* in the “PopGenReport” package (Adamack & Gruber, 2014). Differences in these statistics among populations were tested using the Kruskal–Wallis test. Significance of the pairwise differences in *H*_s_ between populations was tested using the function *gl.test.heterozygosity()* with 1000 replications of re‐randomization. The existence of private alleles (i.e., alleles present in only one population; Kalinowski, 2004) was checked using the function *privates_alleles()* in the “poppr” package (Kamvar et al., 2014), whereas the proportion of shared alleles between pairs of populations was computed with the function *pairwise.propShared()* in the “PopGenReport” package.

We further tested population divergence in the species by computing an overall differentiation index (Fstg) as well as a pairwise genetic differentiation index (Fstp) between pairs of populations, following Weir and Cockerman (1984). Fstg was computed and tested for significance with *overallTest()*, whereas Fstp was obtained with the function *pairwise.wcfst()* in the “STAMPP” package (Pembleton et al., 2013) and tested for significance using 1000 bootstraps across loci.

Finally, we conducted an isolation by distance test to understand the degree to which geographical distance correlates with genetic distance in the species. To accomplish this, a Mantel test was computed between the natural logarithm of geographical distance and the genetic distance quantity (Fstp/[1‐Fstp]) (Gruber et al., 2018; Prior et al., 2020), as implemented in the function *gl.ibd()* in “dartR”. The significance of the Mantel correlation was assessed using 1000 permutations.

#### 2.4.2 Population structure and genetic barrier detection

Population structure was explored using three approaches. First, a hierarchical analysis of molecular variance (AMOVA) was conducted to partition the genetic variation among ecological regions, populations, and individuals. AMOVA was conducted using the function *poppr.amova()* in “poppr”, and the significance of each source of variation was evaluated using a bootstrap method (1000 permutations) through the function *randtest()*. Second, a Bayesian clustering analysis was conducted using STRUCTURE (Pritchard et al., 2000) to determine the number of distinct genetic clusters (*K*) suggested by the accessions in this study. Three independent runs for each test value of *K*, ranging from 2 to 10, were executed with a run length of 10,000 Markov chain Monte Carlo replicates after a burn‐in period of 10,000 iterations, based on a correlated‐allele frequency model. The output was transferred into STRUCTURE HARVESTER (Earl, 2012), where the standard Delta *K* method (Evanno et al., 2005) was used to identify the optimal number of genetic clusters (*K*) supported by the data. Based on the optimal number of clusters identified, a bar plot of assigned genotypes was built using the “Pophelper” package (Francis, 2017). Finally, population structure was investigated via consideration of the phylogenetic relationships among accessions, as evidenced by a neighbor joining model (Saitou & Nei, 1987) based on Tajima‐Nei evolutionary distance (Tajima & Nei, 1984). The phylogeny model was tested using 1000 bootstrap replications in the software MEGA X (Kumar et al., 2018), and the generated tree was exported as a Newick file and edited using the Interactive Tree of Life (ITOL, V.5) (Letunic & Bork, 2021).

To test for the existence of physical/genetic barrier(s) disrupting genetic connectivity among the studied populations, Monmonier's algorithm was implemented in the Barriers software (V.2.2) (Manni et al., 2004).

#### 2.4.3 Nucleotide polymorphism, neutrality statistics, and evolutionary histories of inferred genetic groups

The genetic clusters inferred from the analyses of population structure were used to build new datasets for DNA polymorphism analysis. FASTA files were generated for each genetic cluster using the function *gl2FASTA()* in “dartR” and used for analysis in the software package DnaSP (V.6.12.03) (Rozas et al., 2017). Specifically, the cluster‐based polymorphism data were evaluated by five parameters, namely the number of segregating sites (*S*), the mean number of pairwise nucleotide differences (*K*), the number of haplotypes (*h*), the nucleotide diversity (π), and the haplotype diversity (Hd) (Rozas et al., 2017). The hypothesis of selective neutrality was tested by computing a suite of commonly reported neutrality statistics, namely Tajima's *D*, Fu's *F*s, Achaz's Y*, and Ramos‐Onsins and Rozas's *R*_2_* (Fu & Li, 1993; Ramos‐Onsins & Rozas, 2002; Tajima, 1989). *p*‐Values associated with these indices were obtained through a coalescent simulation based on 1000 bootstrap replications.

## 3 RESULTS

### 3.1 Genotyping

A total of 14,332 raw DArTseq SNPs, called using sequencing data from 322 sampled accessions, were narrowed to a set of 2704 high confidence SNPs (Table S2) after filtering. The polymorphism information content (PIC) of these 2704 markers ranged from 0.09 to 0.49 (mean = 0.41), with a PIC value >0.4 for more than 75% of the markers. The MAF of the final set of markers ranged from 0.051 to 0.5 (mean = 0.32), with MAF >0.3 for more than 70% of markers.

### 3.2 Genetic diversity within and among *S. dulcificum* populations in West Africa

Overall estimates of observed heterozygosity (*H*_o_), expected heterozygosity (*H*_s_), total gene diversity (*H*_t_), and inbreeding (*F*_IS_) in the species were 0.03, 0.15, 0.42, and 0.79, respectively, and all differed significantly from 0 (*p* < 0.001). Population estimates of these statistics are presented in Table 2, along with their associated *p*‐values. The Volta population in the DG consistently exhibited the highest estimates of diversity, while the Western population in the UG block exhibited the lowest estimates of diversity. This signal of higher diversity within the Volta population compared to other populations is further strengthened by the high mean allelic richness exhibited by this population ($A_{r_{\mathrm{VOLT}}}$ = 1.984). In contrast, the lowest allelic richness was observed in the Western population in the UG block ($A_{r_{\mathrm{WEST}}}$ = 1.384) (Table 2).

**TABLE 2:** Genetic diversity indices for the six populations of Sisrè berry plant (*Synsepalum dulcificum*) sampled in this study

Population	*H*_O_	*H*_S_	*F*_IS_	*A*_r_
WEST	0.017	0.080	0.600	1.384
EAST	0.031	0.172	0.816	1.800
VOLT	0.048	0.326	0.830	1.984
MON	0.021	0.096	0.414	1.547
ZOU	0.018	0.071	0.496	1.460
OUE	0.047	0.152	0.628	1.607
*p*	<0.0001	<0.0001	<0.0001	<0.0001

Abbreviations: *H*_O_, Observed heterozygosity; *H*_S_, Expected heterozygosity; *F*_IS_, Inbreeding coefficient; *A*_r_, Mean Allelic richness; WEST, Western population; EAST, Eastern population; VOLT, Volta population; MON, Mono; ZOU, Zou population; OUE, Oueme population.

As shown in Table 3, pairwise comparisons of expected heterozygosity revealed highly significant differences for all population pairs (*p* < 0.0001), except for the pair WEST‐ZOU (*p* < 0.01). No private alleles were detected for any population, but the percentage of shared alleles between pairs of populations ranged widely, from 21.1% (WEST‐ZOU) to 97.1% (MON‐ZOU) (Table 3).

**TABLE 3:** Pairwise differences in expected heterozygosity (lower diagonal) and pairwise proportion of shared alleles (upper diagonal) between sampled populations

Population	WEST	EAST	VOLT	MON	ZOU	OUE
WEST		0.911	0.393	0.220	0.211	0.262
EAST	0.092		0.454	0.270	0.259	0.316
VOLT	0.246	0.150		0.778	0.765	0.815
MON	0.015	0.070	0.230		0.971	0.908
ZOU	0.008 (0.01)	0.100	0.255	0.024		0.909
OUE	0.072	0.019	0.174	0.056	0.080	

*Note*. The values in the lower diagonal are absolute values of differences in expected heterozygosity between pairs of populations, with associated *p*‐values (based on 1000 replications of re‐randomization) in parentheses, only in cases for *p* > 0.0001.

Abbreviations: WEST, Western population; EAST, Eastern population; VOLT, Volta population; MON, Mono; ZOU, Zou population; OUE, Oueme population.

### 3.3 Population divergence and isolation by distance

The calculated global differentiation index (Fst_g_) of 0.63 for this collection of *S. dulcificum* accessions differed significantly from 0 (*p* < 0.0001). All pairwise estimates of the differentiation index (Fst_p_) were also significant, ranging from 0.017 to 0.904 (*p* < 0.0001) (Table 4). The neighboring Mono and Zou populations in the DG were the least differentiated populations (Fst_p_ = 0.017), and the Western (UG) and Zou (Dahomey Gap) populations were found to exhibit the highest level of pairwise divergence (Fst_p_ = 0.904). Fst_p_ ranged from 0.017 to 0.31 for populations within the DG, versus 0.081 to 0.19 in the UG block (Table 4). The Mantel correlation revealed a significant positive correlation (*r* = 0.71, *p =* 0.003) between geographical and genetic distance, indicating a measurable effect of isolation by distance in the West African populations of *S. dulcificum* sampled in this study.

**TABLE 4:** Pairwise differentiation indices (Fstp) between sampled populations of Sisrè berry plant (*Synsepalum dulcificum*) in the study area

Population	WEST	EAST	VOLT	MON	ZOU
EAST	0.081				
VOLT	0.650	0.607			
MON	0.882	0.815	0.276		
ZOU	0.904	0.830	0.310	0.017	
OUE	0.840	0.762	0.162	0.113	0.130

*Note*.All values are significant at the *p* < 0.0001 level.

Abbreviations: WEST, Western population; EAST, Eastern population; VOLT, Volta population; MON, Mono; ZOU, Zou population; OUE, Oueme population.

### 3.4 Population structure and genetic barriers

As shown in the table of hierarchical AMOVA results (Table 5), significant population structure is observed in the species, with 76.0% of the molecular variation attributed to differences between the DG and UG ecological regions. Only 4.0% of the molecular variation could be assigned to differences among populations within ecological regions, while 16.0% was observed among accessions within populations. The percentage of molecular variance attributable to within‐accession variation (4.0%) is essentially equal to that observed among populations.

**TABLE 5:** Table of results from the hierarchical analysis of molecular variance (AMOVA), based on two ecological regions (Upper Guinea and Dahomey Gap), six populations, 322 individual accessions, and 2704 single nucleotide polymorphism (SNP) markers

Source of variation	df	Sum of squares	Mean squares	Variance component	% Variation	*p*
Between ecological regions	1	221724.2	221724.2	746.05	76.0	<0.001
Among populations within regions	4	16799.57	4199.89	39.14	4.0	<0.001
Among accessions within populations	316	111609.2	353.193	157.11	16.0	<0.001
Within accessions	322	12545.5	38.96	38.96	4.0	<0.001
Total	643	362678.5	564.04	982.12	100.0	

**FIGURE 2:**
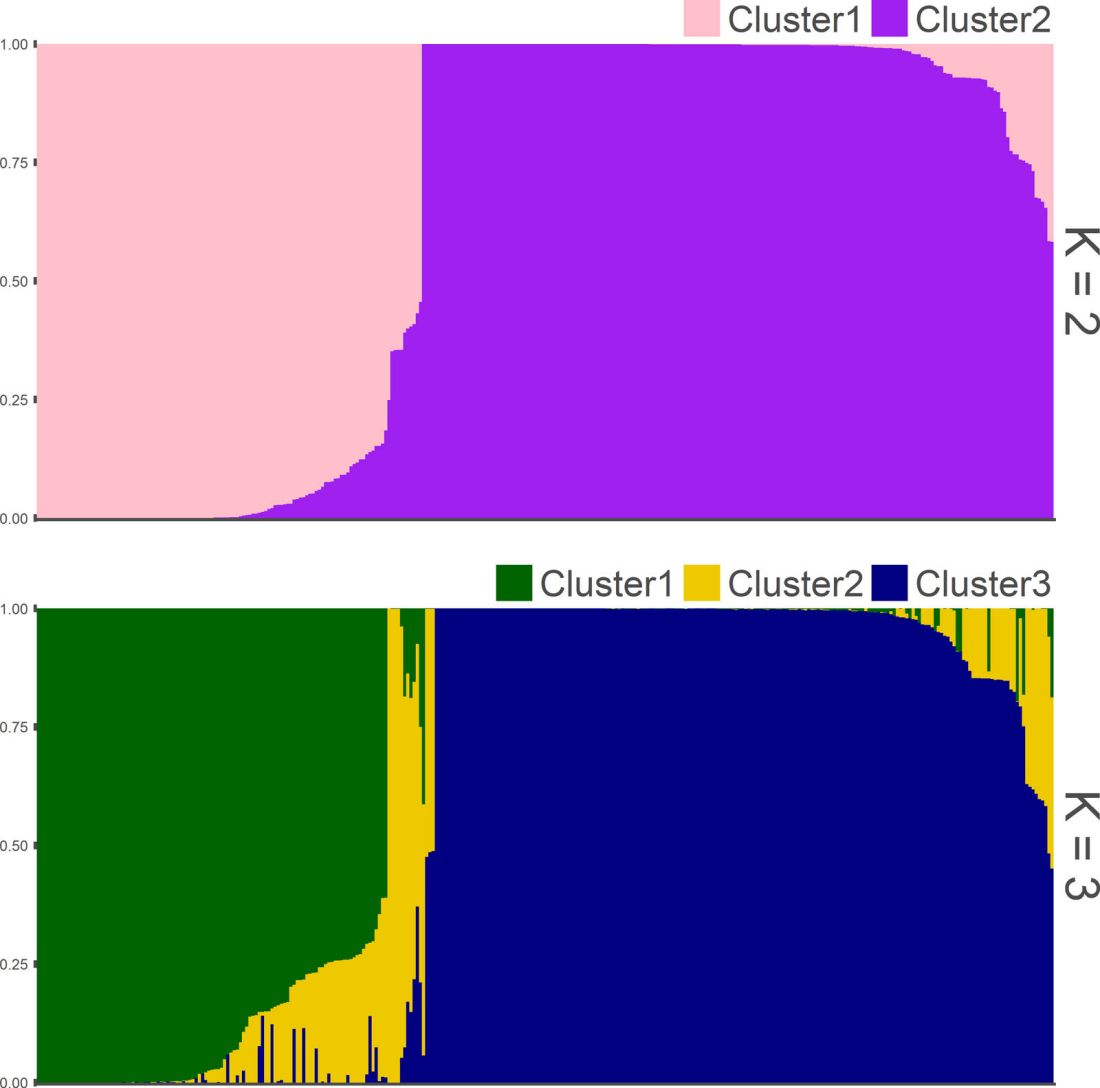
Bar plots showing the structuring pattern of the 322 accessions of Sisrè berry plant (*Synsepalum dulcificum*) collected in the Upper Guinea (UG) and the Dahomey Gap (DG) at both *K* = 2 and *K* = 3, the latter being the optimal number of clusters detected by the STRUCTURE analysis. In the *K* = 2 plot, Cluster 1 = UG genetic group (pink) and Cluster 2 = DG genetic group (purple). In the *K* = 3 plot, the DG group is further split into a Western DG group (gold) and Central DG (dark blue)

Detecting a slightly higher resolution to the West African *S. dulcificum* population structure, the STRUCTURE analysis indicated *K* = 3 as the optimal number of clusters (Figure S1). At *K* = 2, the two observed clusters corresponded perfectly to the UG and DG blocks, echoing the ecological structuring of molecular variation suggested by the AMOVA. Optimization of *K*, however, resulted in a further split of the DG cluster into two sub‐clusters, ultimately leading to the declaration of three distinct genetic groups (Figure S2, Figure 2).

The set of 111 accessions comprising Cluster 1 contains 108 out of the 110 accessions collected in the UG block in Ghana, plus two accessions collected in the VOLT2 sub‐population and one accession from the VOLT3 sub‐population. This cluster represents the UG group. The relatively smaller Cluster 2, hereafter referred to as the Western Dahomey Gap (WDG) group, is composed of 11 accessions, all collected from the VOLT1 sub‐population in the western part of the DG block. Finally, accessions from the MON, ZOU, and OUE populations (Central Dahomey Gap, CDG) clustered with one accession from the EAST population (UG block) and the remaining VOLT2 and VOLT3 accessions to form Cluster 3. This largest cluster, comprising 196 accessions, is hereafter referred to as the CDG genetic group. According to the STRUCTURE analysis, four admixed accessions could not be placed with confidence, including three accessions from the OUE population (DAT347, DAT348, DAT349), disputed by the WDG and CDG groups, and one accession from the EAST population (DAT278), disputed by the UG and WDG groups.

**FIGURE 3:**
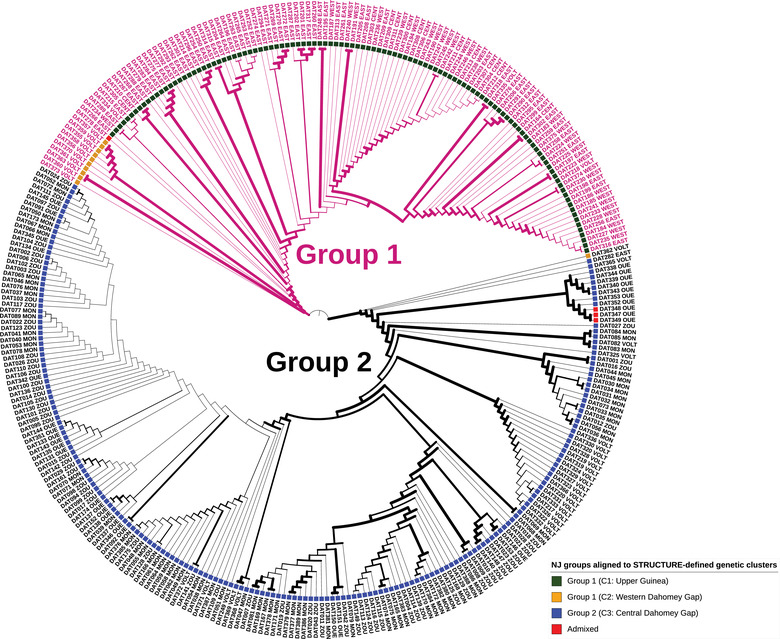
Neighbor joining (NJ) tree‐based phylogenetic relationship among the 322 accessions of Sisrè berry plant (*Synsepalum dulcificum*). Accessions are colored based on their belonging to NJ‐defined groups, whereas associated markers colors indicate STRUCTURE grouping (with red markers indicating admixed accessions). Branch widths are proportional to the bootstrap values (1000 replicates). Each accession name is composed of a three‐digit DAT collection code, followed by the associated sampling population code

**FIGURE 4:**
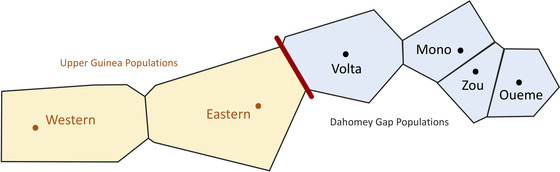
The thick red line indicates the location of a physical barrier (Lake Volta) that effectively disrupts the genetic connectivity between populations of Sisrè berry plant (*Synsepalum dulcificum*) in the Upper Guinea block and those in the Dahomey Gap, as detected by the Barriers (V.2.2.) software. The locations of the dots represent central coordinates for the indicated populations

As shown in Figure 3, the neighbor joining (NJ) tree distinguished two well‐defined groups, in general agreement with the non‐optimal STRUCTURE results for *K* = 2. The further split of the DG group detected by STRUCTURE (*K* = 3) is not, however, reflected with any confidence in the NJ analysis. Based on the NJ tree, the largest group (Group 2, Figure 3) encompasses all the individuals of the STRUCTURE‐inferred CDG genetic group (196 accessions) plus the three admixed accessions from the Oueme population and one accession (DAT362) assigned by STRUCTURE to the WDG group. The other NJ group (Group 1, Figure 3, 122 accessions) mirrors the STRUCTURE‐defined UG and WDG genetic groups, the only difference being one additional admixed accession (DAT278) of UG provenance that was assigned by STRUCTURE to the WDG genetic group. Application of Monmonier's algorithm (Figure 4) provided further evidence of the existence of a physical barrier between the populations in UG and those in DG. Located between the Eastern and Volta populations, this barrier (likely Lake Volta; see Discussion) coincides with the clear first‐order demarcation of population structure reflected in the *K* = 2 STRUCTURE analysis and lends support to the STRUCTURE results of three distinct genetic groups of *S. dulcificum* in the study region. The detailed characteristics of these groups are considered in the next section.

### 3.5 Nucleotide polymorphism and evolutionary history based on inferred genetic groups

The various diversity indices computed for the inferred genetic clusters are presented in Table 6. While mean nucleotide diversity is found to be low in the species (π = 58.36 ± 5 × 10^−5^), it exhibits wide variation across genetic groups. The WDG group exhibits the highest level of nucleotide diversity (π_WDG_ = 156 ± 22 × 10^−5^), and the CDG group presents the lowest (π_CDG_ = 2 ± 0 × 10^−5^). The same trend is observed for haplotype diversity, with the WDG group exhibiting the highest haplotype diversity (Hd_WDG_ = 1.00 ± 0.04) and the CDG the lowest (Hd_CDG_ = 0.76 ± 0.02). In contrast to nucleotide diversity, the average haplotype diversity in the species was found to be high (Hd = 0.9 ± 0.01). In all measures, the UG genetic group consistently presented an intermediate level of diversity (Table 6).

**TABLE 6:** Estimates of nucleotide diversity indices for the three inferred genetic groups of Sisrè berry plant (*Synsepalum dulcificum*) in West Africa: Upper Guinea (UG), Western Dahomey Gap (WDG), and Central Dahomey Gap (CDG)

Genetic group	Ecological region	*S*	*k*	(*π* ± SD) × 10^−5^	*h*	Hd ± SD
UG	Upper Guinea	418	37.74	21 ± 2	62	0.95 ± 0.01
WDG	Dahomey Gap	764	280.62	156 ± 22	10	1.00 ± 0.04
CDG	Dahomey Gap	72	04.08	2 ± 0	36	0.76 ± 0.02

Abbreviations: *S*, number of segregating sites; *k*, average number of pairwise nucleotide differences; *π*, nucleotide diversity; *h*, number of haplotypes; Hd, haplotype diversity.

Regarding neutrality tests (Table 7), Tajima's *D*, Fu's *F*s, and Achaz Y* statistics were found to be consistently negative for the UG and CDG groups but largely positive for the WDG group, except Achaz Y*. While none were significant for the WDG group (*D* = 0.19, *p* = 0.61; *F*s = 1.76, *p* = 0.5; and Y* = −0.08*, p* = 0.47), they were all significant in the CDG group (*D* = −2.03, *p =* 0.002*; F*s = −14.51*, p =* 0.002; and Y* = −1.63*, p* = 0.02), thus suggesting a detectable departure from neutrality in the CDG genetic group. In the UG group, only Tajima's *D* (*D* = −1.75, *p =* 0.01) and Achaz Y* (Y* = −1.94, *p* = 0.005) were found significant. The Ramos‐Onsins and Rozas's *R* ^2^ statistics were low and significant in both the UG and CDG groups but relatively high and not significant in the WDG group (Table 7). As a rule of thumb, positive and significant values of Tajima's *D*, Fu's *F*s, and Achaz Y*, coupled with a large positive R^2^ values, reflect a population bottleneck, while negative and significant values of Tajima's *D*, Fu's *F*s, and Achaz Y*, coupled with small positive and significant *R* ^2^ values, indicate a recent population expansion (Drahun et al., 2021; Ramos‐Onsins & Rozas, 2002; Yingklang et al., 2021). Conversely, non‐significance of Tajima's *D*, Fu's *F*s, and/or Achaz Y* values, coupled with large and non‐significant *R* ^2^, supports the hypothesis of population stability. Likewise, a value of *D* = 0 indicates a selectively neutral population at equilibrium (Tajima, 1989). Taken together, the results suggest recent population expansions of *S. dulcificum* in UG and CDG but relative population stability in WDG.

**TABLE 7:** Neutrality test statistics for the three inferred genetic groups of Sisrè berry plant (*Synsepalum dulcificum*) in the study area

	Ecological zone		Upper Guinea		Dahomey Gap	
Genetic group	UG	*p*	WDG	*p*	CDG	*p*
Tajima's *D* (*D*)	−1.75*	0.01	0.19	0.61	−2.03**	0.002
Fu's FS (*FS*)	−3.582	0.25	1.76	0.50	−14.51**	0.002
Achaz Y* (*Y*)	−1.94**	0.005	−0.08	0.47	−1.63**	0.026
Ramos‐Onsins and Rozas's *R* ^2^	**0.04**	0.007	0.15	0.54	0.03*	0.016

*Note*: Values in bold are significant.

Abbreviations: UG, Upper Guinea; WDG, Western Dahomey Gap; CDG, Central Dahomey Gap.

*, significant at 5%; **, significant at 1%.

## 4 DISCUSSION

Although there have been commendable efforts to conduct systematic phenotypic characterizations of various species within the Sapotaceae (Gwali et al., 2012; Sanou et al., 2006; Tchokponhoué et al., 2020), genomic studies remain rare in the family, particularly those in service to understanding patterns of diversity to support genetic improvement. Recent works on *Vitellaria paradoxa* (Hale et al., 2021), *Argania spinosa* L. Skeels (Khayi et al., 2020; Slimane et al., 2020), and *S. dulcificum* (Yang et al., 2022) are notable in terms of bringing high‐resolution sequencing methods to bear in a family for which SSRs remain the most common marker platform to date (Mouhaddab et al., 2017; Pakhrou et al., 2017; Sanou et al., 2005). This study, the first to employ SNP markers to understand population structure and genetic variation in *S. dulcificum*, lays the groundwork for the systematic, genomics‐assisted conservation and breeding of the species.

Based on the 2704 high‐confidence SNP markers used in this study, an extremely low level of genetic diversity was observed in the species (see Table 2), a result that corroborates the conclusion of Chibuzor et al. (2017), who employed RAPD markers to study the diversity of the species in Southern Nigeria. This low diversity is accompanied by a high inbreeding coefficient (*F*_IS_ = 0.79) indicative of a high levels of fixation that could have resulted from a reduction of effective population size (Szczecińska et al., 2016), especially given that *S. dulcificum* is locally endangered in the study area (Adomou, 2005). This extent of inbreeding could also reflect a predominance of autogamy in the species. The presence of autogamy in *S. dulcificum* was experimentally detected by Tchokponhoué et al. (2017), but whether or not autogamy predominates over allogamy has yet to be clarified. The high inbreeding coefficient also indicates that accessions investigated are near the inbred line state (Gbedevi et al., 2021), an insight that provides impetus to quickly advance breeding population development for agronomic and functional traits of importance in the species. Based on their relatively high expected heterozygosity and mean allelic richness values, the Volta population in particular, and the Eastern and Oueme populations to some extent, stand out as promising sources of genetic material for both breeding and conservation programs.

According to Wright (1978), an Fst value less than 0.05 indicates low differentiation, while values in the ranges 0.05–0.15, 0.15–0.25, and >0.25 suggest moderate, high, and very high differentiation, respectively. Relative to other perennial species (e.g., *Garcinia kola* (Heckel) Fst < 0.09 (Dadjo et al., 2020); *Elaeis* spp. Fst = 0.32 (Pereira et al., 2020); *Macademia* spp. Fst = 0.4 (O'Connor et al., 2019); and *Malus sieversii* Fst < 0.16 (Lebed.) (Richards et al., 2009)), the high singular Fst value (Fstg = 0.63) estimated in this study indicates extremely poor gene flow among West African populations of *S. dulcificum*. Such strong differentiation may in part be due to the significant isolation by distance of the sampled populations. Indeed, as highlighted by the table of pairwise differentiation indices (Table 4), geographically closer populations exhibit comparatively less differentiation. The AMOVA results support this conclusion, highlighting a strong geographical structuring of the molecular variation that aligns with a picture of distance‐mediated, low gene flow in the species. Other factors known to affect the organization of genetic variation in plant species, such as those related to life cycle, reproductive physiology, and seed dispersal mechanisms, may also be relevant (Nybom, 2004). In general, long‐lived outcrossing species are expected to retain higher variation within populations and exhibit high heterozygosity and low differentiation, while annual and selfing species are more variable among populations and exhibit relatively high differentiation and low diversity (Andriamihaja et al., 2021; Kouam et al., 2012). In the long‐lived, presumably predominantly autogamous *S. dulcificum*, our observations match such expectations, particularly in terms of genetic variation largely being partitioned among ecological zones that demarcate mega‐populations of interest in the species.

This hypothesis of ecologically defined groups or mega‐populations is supported by the results of the STRUCTURE analysis, which indicated a primary segregation (at *K* = 2) of the studied accessions and populations into two major gene pools (UG and DG) (see Figure S2), between which gene flow is restricted by the existence of a physical barrier, as evidenced by application of Monmonier's algorithm (Figure 4). Additional evidence for this primary segregation of *S. dulcificum* in West Africa is provided by the table of population pairwise differentiation indices (Table 4), which exhibit a pattern of relatively low values within ecological regions compared to those between regions. The geographical location of the detected barrier, separating the Eastern population of the UG block from the Volta population of the DG block, coincides with Lake Volta (Figure 1), a feature previously pinpointed as a strong biogeographical barrier between the populations in the DG and those in UG of many living organisms (Booth, 1958; Nicolas et al., 2010) and one implicated in the evolutionary divergence of the populations it separates (Moreau, 1969). Indeed, a similar trend of differentiation has been reported between UG and DG populations of other species (e.g., *Pentadesma butyracea* Sabine [Ewédjè, 2012] and *Distemonanthus benthaminus* [Demenou et al., 2016]), but in all cases it is to a lesser extent than that observed here for *S. dulcificum*.

In the end, three distinct genetic pools may be identified in *S. dulcificum*, with the isolation between the UG and DG gene pools maintained and a further split of the relatively more genetically diverse DG gene pool into two potential sub‐populations. Although two widely dispersed UG populations were considered in this study, ultimately all were assigned to a single coherent cluster, echoing the results of other species collected in the region, whether only within Ghana (e.g., *D. benthaminus*; Demenou et al., 2016) or from across several countries (e.g., *Terminalia superba*, collected from Guinea to Ghana [Demenou et al., 2018]). This finding suggests that the connectivity within the Guinean block of the West African forest has been relatively stable, thereby lending support to the hypothesis of the UG comprising an independent and coherent biogeographic region (Linder et al., 2012).

In broad strokes, while STRUCTURE demarcates three groups, NJ demarcates two. There are some minor differences in the clustering patterns of the two analyses that deserves reflections. In both, the Central DG and UG groups are clearly separated. STRUCTURE joins the Western DG to Central DG groups, which is ecologically sound, while NJ joins the Western DG group to the UG group. In the face of this inconsistency, the results of Monmonier's algorithm have been helpful in settling on two distinct genetic clusters, as described above: One in the UG block and one in the DG, corresponding to the WDG and the CDG.

In general, levels of genetic diversity tend to be at their highest in an organism's evolutionary center of origin, with levels decreasing as one moves outward from that center (Rosenbom et al., 2014; Yuan et al., 2008). The analysis of genetic diversity conducted here reveals exceptionally higher levels of nucleotide and haplotype diversity in WDG compared with those of either UG or CDG, even despite the much lower number of accessions assigned to that group.

Although several statistics are useful for inferring organismal evolutionary history, Tajima's *D* is arguably the most well known (Fu, 1997). It is worth recalling that a negative value of *D* reflects a greater prominence of low‐frequency polymorphisms compared to expectation under a neutral model of evolution, thus suggesting a recent demographic expansion. In contrast, a positive value of *D* indicates a loss of both low‐ and high‐frequency polymorphisms, as observed in populations that have undergone a genetic bottleneck (Prior et al., 2020; Tajima, 1989; Yingklang et al., 2021), while a null *D* illustrates a population at equilibrium (neutral selection). Fu's *F*s and Ramos‐Onsins and Rozas’ *R* ^2^ have been shown to be more powerful in detecting population growth, however, with the former being adapted for large populations and *R* ^2^ being more suited to smaller populations (Ramos‐Onsins & Rozas, 2002). As with *D*, a negative and significant Fu's *F*s indicates a recent population expansion, while a positive Fu's *F*s signifies a deficiency of alleles, a typical sign of bottleneck. As for *R* ^2^, lower values indicate populations experiencing expansion (Ramos‐Onsins & Rozas, 2002). Both of these statistics are prone, however, to biased estimation in the presence of sequencing errors, leading to the development of the Achaz Y* statistic (Achaz, 2008) as an alternative.

In light of the various strengths and weaknesses of these statistics, this study considered all four in an effort to infer *S. dulcificum* evolutionary history. Tajima's *D*, Fu's *F*s, and Achaz Y* were all positive and non‐significant in WDG, suggesting that the WDG is a stable population at demographic equilibrium. This conclusion is further supported by the high and non‐significant value of Ramos‐Onsins and Rozas’ *R* ^2^ statistic. Conversely, the clear signature of a recent population expansion is detected in the CDG, as Tajima's *D*, Fu's *F*s, and Achaz Y* were all found to be negative and significant, coupled with a small positive *R* ^2^ statistic (Rosly et al., 2013). In the UG region, significant Tajima's *D*, Achaz Y*, and *R* ^2^ values, combined with a non‐significant negative Fu's *F*s, suggests a more complicated demographic history, characterized by a past population expansion that was possibly geographically restricted within the region (Rosly et al., 2013).

Taken together, the various neutrality statistics paint a portrait of the evolutionary history of the species in which substantively different evolutionary histories exist for the inferred populations. Specifically, the CDG group appears to have experienced a recent population expansion, in contrast to the relatively stable and more diverse WDG group. This is a strong signal that past climate change, manifested through successive vegetation reconfigurations, has markedly impacted the genomic diversity and population structure of *S. dulcificum* in West Africa.

## 5 CONCLUSIONS

Knowledge of molecular variation is crucial for planning rational plant genetic resource conservation and breeding strategies, yet such knowledge is lacking in a great many species, including the economically promising orphan Sisrè berry plant (*S. dulcificum*). Via the application of high‐resolution SNP genotyping to a large and regionally relevant germplasm collection, this study is the first to address this gap in knowledge, providing insight into genetic variation, population structure, and evolutionary history of *S. dulcificum*. Results indicate that overall genetic diversity within the species is low, though highly differentiated populations exist, primarily driven by Lake Volta acting as a geophysical barrier between the DG and the UG gene pools. Such patterning of genetic variation in the species appears to be strongly mediated and structured by ecological conditions, with clear influence from past climate changes. Noticeably, *S. dulcificum* individuals in the UG gene pool underwent a complex demographic change marked by an expansion that was likely restricted to some local regions, while the DG, home to a relatively diverse population of *S. dulcificum*, experienced both a recent demographic expansion (in its Central part) and a demographic equilibrium (in its Western part). Within the DG, the Volta population in particular harbors moderate diversity (*H*_S_ = 0.334) that should be prioritized for collection and evaluation. The high level of fixation within the species presents a unique opportunity to breeding programs interested in identifying superior accessions for fast‐tracked evaluation and release.

The Sisrè berry plant is currently endangered throughout the whole of West Africa, so representatives from all populations should be captured for conservation purposes, but there is urgency for undertaking a thorough germplasm collection in Western DG for the ex situ safeguarding of useful alleles. To facilitate such conservation efforts, the development of an SNP‐based core collection should be considered, particularly as this study has illustrated the viability of low‐cost, high‐resolution genotyping in the species. Finally, the evidence of a physical barrier separating highly differentiated gene pools implies a process of ongoing allopatric speciation within *S. dulcificum*, a possibility that deserves further examination across the entire range of the species, integrating samples not only from the UG and DG regions, but also from the Lower Guinea region, stretching from Nigeria to Central Africa.

## AUTHOR CONTRIBUTIONS

Conceptualization: Dèdéou A. Tchokponhoué, Enoch G. Achigan‐Dako, and Julia Sibiya. Investigation, methodology, data curation, formal analysis, funding acquisition, and project administration: Dèdéou A. Tchokponhoué. Visualization: Dèdéou A. Tchokponhoué and Iago Hale. Validation: Iago Hale and Enoch G. Achigan‐Dako. Writing—original draft: Dèdéou A. Tchokponhoué. Writing—review and editing: Enoch G. Achigan‐Dako, N'Danikou Sognigbé, Daniel Nyadanu, Iago Hale, Alfred O. Odindo, and Julia Sibiya. Supervision: Enoch G. Achigan‐Dako and Julia Sibiya. All authors read the last version of the manuscript and agreed upon its submission.

## CONFLICT OF INTEREST

The authors declare no conflict of interest.

## Supporting information


**Figure S1** Evanno plot indicating Delta K = mean (|L” (K) / sd (L(K)) from STRUCTURE analyses on 322 accessions and 2,704 SNPs in the miracle plant (*Synsepalum dulcificum*).

**Figure S2** Combined bar plots showing population splitting pattern from K = 2 to K = 3 in the miracle plant (*Synsepalum dulcificum*). Pop: Populations sampled; WEST: Western population; EAST: Eastern population, CENT: Central population, VOLT: Volta population, MON: Mono, ZOU: Zou population, and OUE: Oueme population. Ecology: Ecological blocks investigated; UG: upper Guinea and DG: Dahomey gap.

**Box S1**. Detailed protocol used for the Sisrè berry plant DNA extraction

**Table S1**. List of the Sisrè Berry plant accessions and associated geographical coordinates ranges (XLSX file).

**Table S2**. List of the 2,704 SNP markers used in this study (XLSX file).

## Data Availability

All data generated or analyzed during this study are included in this article and its Supporting Information. The raw sequence data will be available on NCBI (NCBI Project # SUB12175840).
