# Supplementary material for: Genome‐wide diversity analysis suggests divergence among Upper Guinea and the Dahomey Gap populations of the Sisrè berry (Syn: miracle fruit) plant (Synsepalum dulcificum [Schumach. & Thonn.] Daniell) in West Africa
Source: Plant Genome. 2023 Jan 20;16(1):e20299. doi: 10.1002/tpg2.20299 (PMC12807384; doi:10.1002/tpg2.20299)
Supplement: Supplementary file 1 [file TPG2-16-e20299-s001.docx]

**Genome-Wide Diversity Analysis Suggests Divergence among Upper Guinea and the Dahomey Gap Populations of the Sisrè Berry (Syn: Miracle Fruit) Plant [*Synsepalum dulcificum* (Schumach. & Thonn*.*) Daniell] in West Africa**

Dèdéou A. Tchokponhoué^1,2*^, Enoch G. Achigan-Dako^2^, N’Danikou Sognigbé^2,3,4^, Daniel Nyadanu^5^, Iago Hale^6^, Alfred O. Odindo^1^ and Julia Sibiya^1^

**Supplemental file**

**Box S1**. Detailed protocol used for the Sisrè berry plant DNA extraction.

The DNA extraction was based on NucleoMag® Plant procedure. Indeed, 50 mg of dried leaf sample was homogenized using microtubule strips in a mixer mill. 500 μL of a lysis Buffer MC1 was added to the homogenized leaf sample before shaking for 20 s and spinning at 1,500 x g for 30 s. The homogenized mixture was incubated for 30 min at 56 °C. The lysate was cleared by centrifugation at a full speed of 5,800 x g for 20 min. Afterwards, 400 μL of the cleared lysate was transferred to a square-well block to which 30 μL of NucleoMag® C-Beads and 400 μL of a binding Buffer MC2 were added to each well for the DNA binding to NucleoMag® C-Beads. The magnetic beads were afterwards separated against the side of the wells by placing the squarewell block on the NucleoMag® SEP magnetic separator. After all the beads were attracted to the magnets, the supernatant was gently removed and discarded from the opposite side of the well by pipetting. The Square-well Block was then removed from the NucleoMag® SEP magnetic separator and 600 μL of a wash Buffer MC3 was added to each well and shaken for 5 min until complete resuspension of the beads. This washing process was repeated two more times, the first time with 600 μL of a wash Buffer MC4 and the second time with 600 μL of 80% ethanol. A last washing step was conducted, to remove traces of ethanol, this time by leaving the Square-well Block on the NucleoMag® SEP magnetic separator. To that, 600 μL of a wash Buffer MC5 was added to each well and the mixture incubated for 50 s while the beads are still attracted to magnets. The supernatant was afterwards aspirated and discarded. Finally, the Square-well Block was removed from the NucleoMag® SEP magnetic separator and 150 μL of an elution buffer MC6 was added to each well of the Square-well Block and the beads were resuspended by shaking for 5–10 min at 56 °C. The magnetic beads were ultimately separate by placing the Square-well Block on the NucleoMag® SEP magnetic separator and waiting 3 min until all the beads have been attracted to the magnets. The supernatant containing the purified genomic DNA was then transferred to the elution plate.
